# Modeling of antibody responses to COVID-19 vaccination in patients with rheumatoid arthritis

**DOI:** 10.1038/s41598-024-51535-4

**Published:** 2024-01-16

**Authors:** Yun Kyu Kim, Yunhee Choi, Ji In Jung, Ju Yeon Kim, Mi Hyeon Kim, Jeffrey Curtis, Eun Bong Lee

**Affiliations:** 1 Division of Rheumatology, Department of Internal Medicine, Seoul National University Hospital, Seoul, Republic of Korea; 2 Medical Research Collaborating Center, Seoul National University Hospital, Seoul, Republic of Korea; 3 Division of Clinical Immunology and Rheumatology, University of Alabama at Birmingham, Birmingham, USA; 4 Department of Molecular Medicine and Biopharmaceutical Sciences, Graduate School of Convergence Science and Technology, Seoul National University, Seoul, Republic of Korea; 5 Division of Rheumatology, Department of Internal Medicine, Seoul National University College of Medicine, 101 Daehak-ro, Jongno-gu, Seoul, 110-744 Republic of Korea

**Keywords:** Immunology, Rheumatology

## Abstract

To construct a model of the antibody response to COVID-19 vaccination in patients with rheumatoid arthritis (RA), and to identify clinical factors affecting the antibody response. A total of 779 serum samples were obtained from 550 COVID-19-naïve RA patients who were vaccinated against COVID-19. Antibody titers for the receptor binding domain (anti-RBD) and nucleocapsid (anti-N) were measured. The time from vaccination, and the log-transformed anti-RBD titer, were modeled using a fractional polynomial method. Clinical factors affecting antibody responses were analyzed by a regression model using generalized estimating equation. The anti-RBD titer peaked at about 2 weeks post-vaccination and decreased exponentially to 36.5% of the peak value after 2 months. Compared with the first vaccination, the 3rd or 4th vaccinations shifted the peaks of anti-RBD antibody response curves significantly upward (by 28-fold [4–195] and 32-fold [4–234], respectively). However, there was no significant shift in the peak from the 3rd vaccination to the 4th vaccination (p = 0.64). Multivariable analysis showed that sulfasalazine increased the vaccine response (by 1.49-fold [1.13–1.97]), but abatacept or JAK inhibitor decreased the vaccine response (by 0.13-fold [0.04–0.43] and 0.44-fold [0.26–0.74], respectively). Age was associated with lower ln [anti-RBD] values (coefficient: − 0.03 [− 0.04 to − 0.02]). In conclusion, the anti-RBD response of RA patients peaked at 2 weeks after COVID-19 vaccination, and then decreased exponentially, with the maximum peak increase observed after the 3rd vaccination. The antibody response was affected by age and the medications used to treat RA.

## Introduction

COVID-19 vaccinations have successfully reduced the incidence of COVID-19 infections, as well as the severity of symptoms^1–5^. The titer of protective antibodies is an important marker of protection against infection. Therefore, several studies have investigated the kinetics of vaccine responses in the general population^6,7^. The study of Virus Watch cohort, which examined 24,997 samples from 9492 individuals to measure the level of SARS-CoV2 antibodies targeting the spike protein (anti-S) after two vaccine doses, showed that anti-S levels peaked at 3–4 weeks after vaccination, and declined thereafter^8^. This rapid decline of titer caused the Centers for Disease Control (CDC) to recommend repeated booster vaccinations. Therefore, a comprehensive understanding of the dynamics of humoral immunity is essential for planning effective vaccination strategies.

Patients with rheumatoid arthritis (RA) have underlying immune dysregulation and are often taking immunosuppressive medications; therefore, the antibody response patterns are more complex in them^9^. Few studies have investigated vaccine response kinetics in RA patients in a real-world situation. Furthermore, it is not clear whether repeated vaccination can induce similar levels of immunogenicity after each booster. Therefore, we aimed to construct an antibody response model to monitor the dynamics of the humoral immune response to COVID-19 vaccination in patients with RA.

## Methods

### Study design

The purpose of the study was to examine the antibody response in RA patients vaccinated against COVID-19 and to identify clinical factors affecting the antibody response in a real-world setting. In this study, the type of vaccination and the intervals between vaccinations were heterogeneous among the patients; this is because COVID-19 vaccinations were administered as part of routine clinical practice. The primary analysis involved measurement of antibody titers from RA patients that received the BNT162b2, mRNA-1273, ChAdOx1, or Ad26.COV2.S vaccines. All vaccines were monovalent as no bivalent vaccines were available at the time. A patient was censored if he/she was infected with COVID-19 during follow-up. Antibody response curves were constructed after the 1st, 2nd, 3rd, and 4th vaccinations; these were based on the anti-RBD antibody titers measured after each vaccination. To identify factors that contribute to a peak response, the antibody response for each patient was modeled based on their individual clinical factors.

The study was carried out in accordance with the Declaration of Helsinki, and was approved by the Institutional Review Boards of Seoul National University Hospital (IRB No. 2205-060-1322).

### Patients

South Korea experienced two major peaks of COVID-19 infection: Feb 2022 and Sep 2022. Two groups of RA patients attending Seoul National University Hospital (SNUH), a nationwide tertiary referral center in South Korea, were enrolled before the COVID-19 peaks occurred. Group 1 comprised RA patients enrolled in an influenza vaccination study between October 6 and November 3, 2021 (IRB No. 2109-020-1252). The original study was a randomized controlled trial to evaluate the vaccination response by comparing a 1-week versus 2-week temporary discontinuation of MTX after influenza vaccination. According to the enrollment criteria, all RA patients in Group 1 had taken stable dose of methotrexate over the preceding 6 weeks of influenza vaccination. Serial serum samples were obtained at 0, 4, and 16 weeks after the influenza vaccination. Group 2 comprised patients who participated in the SNUH RA cohort study between January 1 and June 3, 2022 (IRB No. 2105-085-1219). The study was a cohort study to monitor disease activity and treatment response. For these patients, sera were obtained once at the start of participation. The samples analyzed in the study were all archived samples, not additional blood draws. Informed consent was obtained from all participants to use their samples for further study at the time of the enrollment.

Among the enrolled patients, only those with an available vaccination history and who were naïve to COVID-19 infection were included in data analysis. The exclusion criteria were as follows: (1) self-reported or a Korea Disease Control and Prevention Agency (KDCA) record of COVID-19 infection before sampling; (2) positive for anti-nucleocapsid (anti-N) antibodies; (3) did not receive a COVID-19 vaccination before sampling.

### Demographics and patient characteristics

Patient demographics, comorbidities, and concurrent immunosuppressive medications were obtained from electronic medical records. Comorbidities included diabetes mellitus, hypertension, chronic liver disease, chronic kidney disease, and history of tuberculosis. Concomitant medications were defined as those prescribed within 3 months of blood sampling. These included glucocorticoids (GCs), methotrexate (MTX), hydroxychloroquine, sulfasalazine (SSZ), leflunomide, tacrolimus, tumor necrosis factor alpha inhibitors (TNFi), tocilizumab, abatacept (ABA), Janus kinase inhibitors (JAKi), and rituximab (RTX).

### COVID-19 vaccination and COVID-19 infection

Since February, 2021 in South Korea, COVID-19 vaccinations have been mandatory in accordance with the national guidelines. The type of vaccine and intervals between vaccinations were decided by the government. The first approved vaccines were BNT162b2, mRNA-1273, ChAdOx1, and Ad26.COV2.S. With the exception of Ad26.COV2.S, all primary vaccinations required a follow-up 2nd dose after 3–12 weeks. Cross-vaccination was allowed. In December 2021, the 3rd dose of vaccine was administered (i.e., an interval of 2–3 months after the previous vaccination). In April 2022, a 4th dose was recommended (i.e., an interval of 4 months from the previous vaccination). The vaccination history of each patient was listed by the KDCA. Any PCR-proved COVID-19 infection should be reported to the KDCA through the regional infection center or a local clinic.

Information on the dose, date and the type of COVID-19 vaccination, was obtained from the patients and cross-checked with the data from KDCA. Previous COVID-19 infection was reported by the patients, and confirmed by an infection certificate from KDCA and a positive anti-nucleocapsid (anti-N) antibody test. When the patient tested positive in the self-antigen test but did not undergo a formal diagnostic test for any reason, the patient was considered as a “positive infection” case and excluded.

### Measurement of SARS-CoV-2 antigen-specific antibodies

The titer of IgG antibodies specific for the SARS-CoV-2 receptor binding domain of spike 1 protein (anti-RBD) was measured in stored serum samples using a chemiluminescence microparticle immunoassay (Abbott, USA). The anti-RBD ranged from 21 to 40,000 AU/mL. A value < 21 or > 40,000 AU/mL was documented as 20 or 44,000 AU/mL, respectively. Anti-RBD antibodies represent the humoral response to COVID-19 vaccination^10^.

In addition, the titer of anti-N antibodies was measured using an electrochemiluminescence immunoassay (Roche, Germany). Anti-N antibody titers above the cut-off value of 1.00 AU/mL denoted a previous natural COVID-19 infection^11^.

### Sensitivity analysis

Since the two studies (influenza vaccination study and SNUH RA cohort study) used in this model were not specifically designed for modeling of antibody response to COVID-19 vaccination, vaccination and sampling schedules were heterogenous among individuals. Therefore, we performed several sensitivity analyses to reconfirm our results.

First, we estimated the change of the log anti-RBD titer over time for the subjects who received the same type of vaccination. Second, the group 1 (influenza vaccination study) and group 2 (SNUH RA cohort study) were separately analyzed to investigate the clinical factors affecting anti-RBD titer and to reveal time-course of anti-RBD antibodies following vaccination.

### Statistical analysis

The characteristics of the subjects were expressed as mean (standard deviation) for continuous variables and numbers (percentage) for categorical variables. The anti-RBD titer was log-transformed to improve normality. The second-degree fractional polynomials which covers wide range of curve shapes were applied since the pattern of change in the log anti-RBD titer over time is unknown and may not be linear^12^. The change of the log anti-RBD titer over time was determined in the fractional polynomial regression while adjusting the vaccination dose. Robustness of the curve was confirmed with adjustment of clinical factors affecting log anti-RBD titer: age, ABA use JAKi use, SSZ use, and the vaccination dose. Then clinical variables related to humoral responses to COVID-19 vaccination were determined. A regression model using a generalized estimating equation was applied to account for the correlation among anti-RBD titers among the subjects. Since only 22% subjects (120 out of 550) have two or three antibody responses, we chose the population average model, instead of a subject specific estimate. The exchangeable variance structure was applied because the interval between repeated measurements were various from subject to subject, and only 20% of the subjects had three anti-RBD titer. However, the mixed effect model was used to estimate the change of the log anti-RBD for Group1 subjects (from whom serial samples were obtained). The time and vaccination dose were fixed effects and the subject was a random effect. The linear assumption of continuous variables was checked using scatter plot and locally weighted scatterplot smoothing with clinical knowledge of relationship with log anti-RBD titer. The significant variables at 0.1 significance levels in the univariable analysis were considered for the multivariable model. The forward variable selection method was used to detect significant clinical variables affecting the log anti-RBD titer. The all two-way interaction terms were tested in the multivariable model one by one at 0.01 level of significance. The goodness of fit for the multivariable model was measured using R-square.

Statistical analysis was performed using R (version 4.3.1; R Foundation for Statistical Computing) and SAS software (version 9.4; SAS Institute).

## Results

### Clinical characteristics

**Figure 1:**
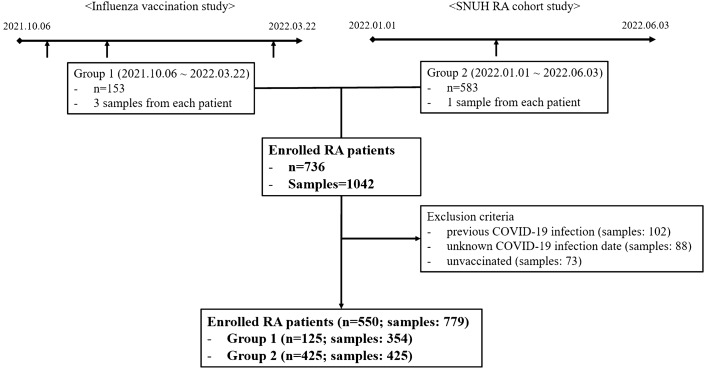
Flow of the patient inclusion.

A total of 736 patients (1042 serum samples) were enrolled. Among the 736 patients, 153 (459 samples) were included in Group 1 (influenza vaccination study) and 583 (583 samples) were included in Group 2 (SNUH RA Cohort). After excluding patients who did not fulfill the inclusion criteria, 550 patients with 779 samples (125 patients (354 samples) from Group 1, and 425 patients (425 samples) from Group 2) were included in the final analysis (Fig. 1).

The clinical characteristics of the included patients are shown in Table 1. The majority of samples were obtained after the 2nd or 3rd vaccinations (39.4% and 56.7%, respectively). The type of vaccine is listed in Supplementary table 1.

**Table 1:** Characteristics of the included patients.

	Group 1 (N = 125)	Group 2 (N = 425)	Total (N = 550)
Female sex, n (%)	107 (85.6)	365 (85.9)	472 (85.8)
Age, years	60.8 (11.9)	64.6 (12.4)	63.8 (12.4)
BMI, kg/m^2^	22.9 (3.6)	23.6 (3.2)	23.4 (3.4)
DM, n (%)	12 (9.6)	48 (11.3)	60 (11.0)
Hypertension, n (%)	41 (32.8)	112 (26.5)	153 (27.9)
History of tuberculosis, n (%)	9 (7.2)	18 (4.3)	27 (4.9)
Chronic liver disease, n (%)	0 (0.0)	25 (5.9)	25 (4.6)
Chronic kidney disease, n (%)	2 (1.6)	19 (4.5)	21 (3.8)
RA duration, years	12.3 (7.6)	9.9 (6.1)	10.5 (6.6)
Glucocorticoids use, n (%)	64 (51.2)	221 (52.0)	285 (51.8)
Prednisolone equivalent, mg	2.1 (2.4)	2.24 (2.6)	2.2 (2.6)
Methotrexate, n (%)	125 (100.0)	267 (62.8)	392 (71.3)
Hydroxychloroquine, n (%)	29 (23.2)	150 (35.3)	179 (32.6)
Sulfasalazine, n (%)	7 (5.6)	57 (13.4)	64 (11.6)
Leflunomide, n (%)	22 (17.6)	75 (17.7)	97 (17.6)
TNF inhibitors, n (%)	10 (8.0)	33 (7.8)	43 (7.8)
Tocilizumab, n (%)	3 (2.4)	7 (1.7)	10 (1.8)
Abatacept, n (%)	2 (1.6)	4 (0.9)	6 (1.1)
Rituximab, n (%)	0 (0.0)	1 (0.2)	1 (0.2)
JAK inhibitor, n (%)	6 (4.8)	31 (7.3)	37 (6.7)
Tacrolimus, n (%)	3 (2.4)	7 (1.7)	10 (1.8)
Vaccination dose^a^, n (%)			
1	2 (1.6)	6 (1.4)	8 (1.5)
2	30 (24.0)	64 (15.1)	94 (17.1)
3	93 (74.4)	339 (79.8)	432 (78.5)
4	0 (0.0)	16 (3.8)	16 (2.9)

Data are shown as mean (SD) unless otherwise specified.

*BMI*, body mass index; *DM*, diabetes mellitus; *JAK*, janus kinase ; *RA*, rheumatoid arthritis; *TNF*, tumor necrosis factor.

^a^Total number of serum samples for the 1st vaccination was 14 (1.8%), the number for the 2nd vaccination was 307 (39.41%), the number for the 3rd vaccination was 442 (56.74%), and the number for the 4th vaccination was 16 (2.05%).

### Dynamics of the anti-RBD titer over time post-vaccination

The anti-RBD titer showed time-dependence according to the vaccination dose by the formula below.

ln(RBD) = 8.12–1.96·ln(t)-2.45·t^−0.5^ + 1.95·(V_2_) + 3.47·(V_3_) + 3.47·(V_4_) (V_i_ = 1, when vaccination dose is i).

**Figure 2:**
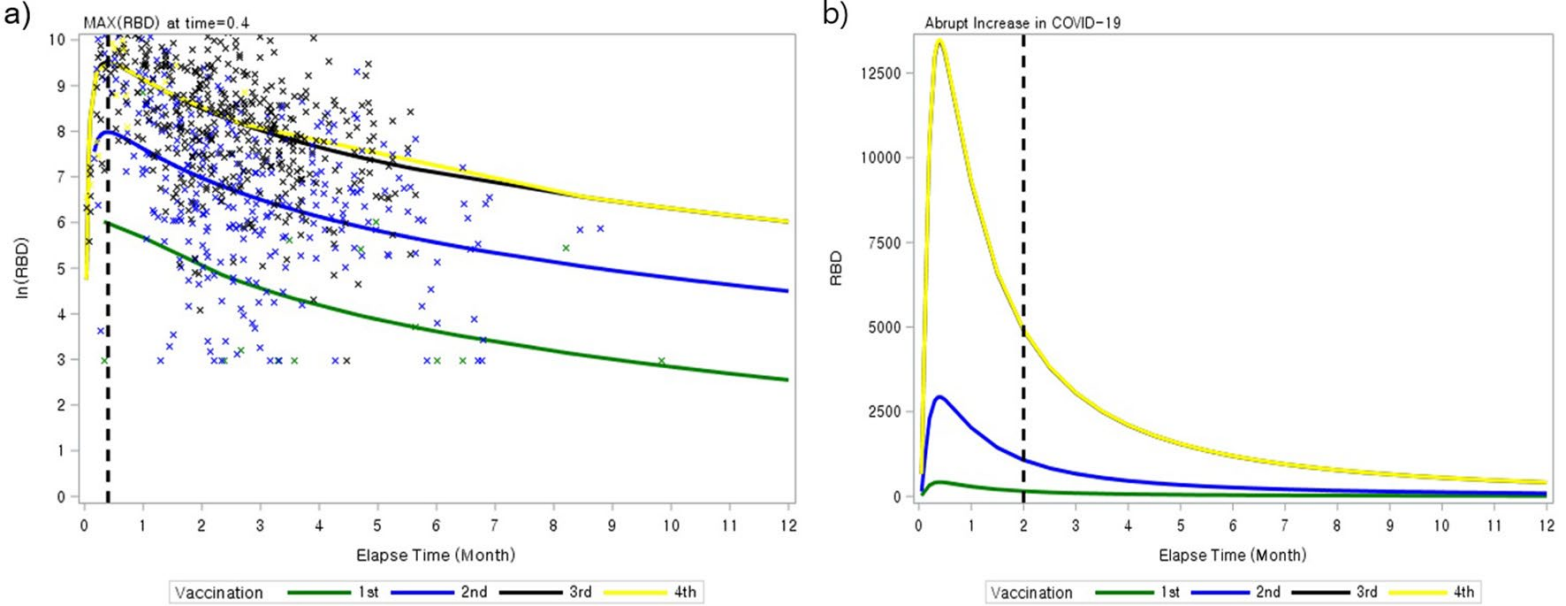
Anti-RBD titer curve after each dose of COVID-19 vaccination. (**a**) ln (anti-RBD antibody) value (**b**) anti-RBD antibody value.

The anti-RBD titer increased exponentially, peaking at 12 days post-vaccination, and followed a log-linear decline to 36.5% of the peak value after 2 months (Fig. 2). When age, use of SSZ, ABA and JAKI were adjusted, the 3rd or 4th vaccinations led to a significant increase in the anti-RBD antibody response compared with the first vaccination (by 28-fold [4–195] and 32-fold [4–234], respectively). However, there was no significant difference between the peak titers measured after the 3rd and 4th vaccinations (p = 0.64).

### Clinical factors affecting the humoral response to COVID-19 vaccination

Risk factors for a reduced humoral response to COVID-19 vaccination are listed in Table 2. The interaction between age and use of JAK inhibitor and the interaction between age and vaccination dose were significant at 0.01 level of significance. However, the interactions did not significantly change the interpretation of the relationship with humoral response. Hence, they were excluded in the multivariable model.

**Table 2:** Regression model for ln (anti-RBD) after COVID-19 vaccination.

		Univariable^a^			Multivariable^c^		
		Coefficient	95% CI	p-value^b^	Coefficient	95% CI	p-value
Female sex		0.17	− 0.22 to 0.55	0.393	–	–	–
Age (per 10 year)		− 0.21	− 0.30 to − 0.12	< 0.001**	− 0.29	− 0.37 to − 0.21	< 0.001**
BMI		− 0.03	− 0.08 to 0.02	0.202	–	–	–
DM		0.11	− 0.27 to 0.50	0.568	–	–	–
Hypertension		− 0.38	− 0.69 to − 0.07	0.018**	–	–	–
History of tuberculosis		− 0.61	− 1.35 to 0.12	0.100*	–	–	–
Chronic liver disease		0.31	− 0.16 to 0.78	0.196	–	–	–
Chronic kidney disease		0.68	0.07 to 1.29	0.030**	–	–	–
RA duration		− 0.04	− 0.06 to − 0.01	0.001**	–	–	–
Glucocorticoid use		− 0.12	− 0.38 to 0.14	0.357	–	–	–
Prednisolone equivalent		− 0.06	− 0.11 to 0.00	0.035**	–	–	–
Methotrexate		− 0.23	− 0.52 to 0.05	0.105	–	–	–
Hydroxychloroquine		0.01	− 0.28 to 0.29	0.965	–	–	–
Sulfasalazine		0.45	0.07 to 0.84	0.022**	0.40	0.12 to 0.68	0.005**
Leflunomide		0.00	− 0.37 to 0.37	0.988	–	–	–
Tacrolimus		− 0.14	− 0.92 to 0.63	0.718	–	–	–
TNF inihibitors		0.29	− 0.16 to 0.73	0.208	–	–	–
Tocilizumab		− 0.03	− 0.78 to 0.72	0.939	–	–	–
Abatacept		− 2.07	− 3.14 to − 0.99	< 0.001**	− 2.07	− 3.30 to − 0.84	0.001**
JAK inhibitor		− 0.88	− 1.52 to − 0.24	0.007**	− 0.82	− 1.34 to − 0.31	0.002**
Rituximab		− 2.28	− 2.41 to − 2.15	< 0.001**	–	–	–
Time from vaccination (month)	ln(t)	− 1.96	− 2.37 to − 1.54	< 0.001**	− 1.90	− 2.29 to − 1.50	< 0.001**
	t^−0.5^	− 2.45	− 3.30 to − 1.60	< 0.001**	− 2.40	− 3.21 to − 1.60	< 0.001**
Vaccination dose	1						
	2	1.95	− 0.26 to 4.15	0.084*	1.77	− 0.16 to 3.71	0.072*
	3	3.47	1.26 to 5.68	0.002**	3.33	1.40 to 5.27	0.001**
	4	3.47	1.20 to 5.74	0.003**	3.46	1.48 to 5.45	0.001**

*BMI*, body mass index; *DM*, diabetes mellitus; *JAK*, janus kinase ; *RA*, rheumatoid arthritis; *TNF*, tumor necrosis factor.

^a^Covariates that showed a relevant association (p < 0.1) with the outcome in the univariable analysis were included in the multivariable analysis.

^b^Significance are shown as ‘*’ for ‘p < 0.1’ and ‘**’ for ‘p < 0.05’.

^c^The R-square for the multivariable model was 0.469.

In RA patients, older age led to a significant reduction in the humoral response. Multivariable analysis identified older age as being associated with a lower ln [anti-RBD] (coefficient: − 0.03 [− 0.04 to − 0.02]). This would mean, for example, that the humoral response in a 70-year-old patient is only 25% of that in a 20-year-old patient.

Interestingly, SSZ use increased the humoral response. Multivariable analysis revealed that patients who used SSZ had a higher anti-RBD titer (by 1.49-fold [1.13–1.97]) than those who did not. However, use of ABA or JAKi decreased the anti-RBD titer (by 0.13-fold [0.04–0.43] and 0.44-fold [0.26–0.74], respectively). Other immunosuppressants such as GC, MTX, and RTX had no significant effect on the humeral response. The R-square for the multivariable model was 0.469.

### Sensitivity analysis

Even though the effect of the 4th vaccination could not be analyzed fully given sparse data, the antibody response to COVID-19 vaccination was similar, anti-RBD reached its peak at about 2 weeks and decreased exponentially, when we restricted the analysis to those who received the same type of vaccine (Supplementary Fig. 1).

And, when we analyzed Group 1 and 2 separately, patient’s age, time from vaccination and vaccination dose remained significant in multivariable analysis (Supplementary Tables 2, 3). ABA and JAKi were not simultaneously significant in multivariable analysis of both groups, but they showed negative correlation in univariable analysis of both groups. Anti-RBD titer reached its peak at about 2 weeks and decreased exponentially in both groups (Supplementary Figs. 2, 3), consistent with the pattern from the whole data (Fig. 2).

## Discussion

In this study, we modeled anti-RBD antibody responses in RA patients after COVID-19 vaccination and identified clinical factors affecting these antibody responses. After vaccination, anti-RBD antibody titers increased exponentially to reach a peak at about 2 weeks; this was followed by decline along a log-linear course. This trend was consistent with that of the general population which peaked at 2–4 weeks after vaccination and declined log-linearly^7,8,13,14^. Patients who received a booster vaccination showed a significantly higher humoral response curve than those who only received one dose. However, there was no significant difference in peak humoral response between the 3rd and 4th vaccinations. These results are consistent with those of a recent report of healthy volunteers showing that the peak anti-RBD antibody response to the 4th vaccination was inferior to that after the 3rd vaccination^15^. Thus, the data suggest that 3rd vaccination would be sufficient to induce maximum immunogenicity in RA patients. Further studies in RA patients are needed to assess the additional benefits provided by further vaccinations.

In multivariable analysis, patients with RA showed negative correlation between age and humoral response, which was consistent with the result of the general population in previous studies^16,17^. Also, certain immunosuppressive medications (ABA and JAKi) were associated with a weaker humoral response to vaccination. This suggests that RA patients who are older or taking immunosuppressive medications may be less protected against SARS-Cov-2 infections, even when vaccinated according to current schedules. For these patients, a special strategy may be needed, which may include increasing the vaccine dose, increasing the frequency of boosting, or an adequate period of discontinuation of immunosuppressive medications. We also found that patients taking SSZ had a significant increase in anti-RBD titer after vaccination. Further studies are needed to confirm these results. Interestingly, those taking MTX did not show a significant decrease in the humoral response. This might be because most patients enrolled in our study were recommended to discontinue MTX for 1 or 2 weeks post-vaccination according to our previous study on influenza vaccination^18^. GCs did not suppress the humoral response either; however, the dose GCs in this study population was rather low (mean prednisolone equivalent of 4.2 mg/day).

This study has several limitations. First, the number of the patients vaccinated only once or four times was lower than the number vaccinated 2 or 3 times. Hence, the antibody response patterns for 1st or 4th vaccination were not fully described. However, the antibody response patterns differed according to the number of vaccinations (Fig. 2). Second, the total number of patients may not be enough to analyze clinical factors affecting the antibody response; however, the risk factors identified in this study (aging and use of ABA and JAKi) are consistent with findings from published studies^16,19,20^. Third, the two studies used in our model were not specifically designed to evaluate humoral response to COVID-19 vaccination. Therefore, different combinations of vaccines were used, and the timing of biospecimen collection was somewhat variable. Since the type of vaccine was determined by the government, the same vaccines were not used consistently. However, all of the vaccines targeted the same RBD protein, and our results represent a real-world situation in which different combinations are used. Finally, biospecimens were serially collected in only one third of the patients, so our findings may be skewed by the sampling schedule.

In conclusion, anti-RBD response to COVID-19 vaccination in patients with RA peaked at about 2 weeks post-vaccination and decreased exponentially. The peak antibody response increased significantly up to the 3rd vaccination, but no additional benefit was seen after the 4th vaccination. The antibody response is affected by age and by concurrent medications used to treat RA.

### Supplementary Information

Supplementary Information.

## Data Availability

The datasets generated and analyzed during the current study are available from the corresponding author on reasonable request.
