# Supplementary material for: Modeling of antibody responses to COVID-19 vaccination in patients with rheumatoid arthritis
Source: Sci Rep. 2024 Jan 16;14:1335. doi: 10.1038/s41598-024-51535-4 (PMC10791674; doi:10.1038/s41598-024-51535-4)

Modeling of antibody responses to COVID-19 vaccination in patients with rheumatoid arthritis

Yun Kyu Kim*[1], Yunhee Choi*[2], Ji In Jung[1], Ju Yeon Kim[1], Mi Hyeon Kim[1], Jeffrey Curtis[3], Eun Bong Lee[1,4]

[1] Division of Rheumatology, Department of Internal Medicine, Seoul National University Hospital, Seoul, Republic of Korea

[2] Medical Research Collaborating Center, Seoul National University Hospital, Seoul, Republic of Korea

[3] Division of Clinical Immunology and Rheumatology, University of Alabama at Birmingham, USA

[4]Department of Molecular Medicine and Biopharmaceutical Sciences, Graduate School of Convergence Science and Technology, Seoul National University, Seoul, Republic of Korea.

*Yun Kyu Kim and Yunhee Choi are equal contributors to this work and designated as co-first authors.

***Reprint requests and correspondence to:**

Eun Bong Lee, MD., PhD.

Division of Rheumatology, Department of Internal Medicine

Seoul National University College of Medicine

101 Daehak-ro, Jongno-gu, Seoul, 110-744, Republic of Korea

E-mail: leb7616@snu.ac.kr

(Supplementary table 1) Samples according to the final vaccination type

Final vaccination type	Sample (N=779)
ChAdOx1	119 (15.3)
mRNA-1273	126 (16.2)
BNT162b2	533 (68.4)
Ad26.COV2.S	1 (0.0)

Numbers in parentheses = %.

(Supplementary table 2) Regression model for ln (anti-RBD) after COVID-19 vaccination in patients from Group 1

		Univariable			Multivariable	
	coefficient	95% CI	p-value	coefficient	95% CI	p-value
Female sex	0.37	-0.37 to 1.10	0.331	-	-	-
Age (per 10 year)	-0.59	-0.79 to -0.40	<.001	-0.42	-0.61 to -0.22	<.001
BMI	-0.07	-0.14 to 0.00	0.058	-	-	-
DM	0.03	-0.87 to 0.93	0.944	-	-	-
Hypertension	-0.84	-1.38 to -0.30	0.002	-0.61	-1.09 to -0.13	0.013
History of tuberculosis	-0.76	-1.75 to 0.24	0.137	-	-	-
Chronic liver disease	-	-	-	-	-	-
Chronic kidney disease	-0.89	-2.94 to 1.16	0.393	-	-	-
RA duration	-0.06	-0.09 to -0.02	0.001	-	-	-
Glucocorticoid use	0.02	-0.51 to 0.54	0.947	-	-	-
Prednisolone equivalent	-0.10	-0.20 to 0.01	0.076	-	-	-
Methotrexate	-	-	-	-	-	-
Hydroxychloroquine	-0.01	-0.63 to 0.61	0.983	-	-	-
Sulfasalazine	0.70	-0.45 to 1.84	0.232	-	-	-
Leflunomide	-0.32	-1.01 to 0.36	0.353	-	-	-
Tacrolimus	0.57	-1.12 to 2.25	0.507	-	-	-
TNF inhibitors	-0.005	-0.98 to 0.97	0.992	-	-	-
Tocilizumab	0.66	-1.02 to 2.35	0.437	-	-	-
Abatacept	-0.60	-2.66 to 1.46	0.566	-	-	-
JAK inhibitor	-1.37	-2.60 to -0.14	0.029	-	-	-
Rituximab	-	-	-	-	-	-
Time from vaccination (month) ln(t)	-1.99	-2.30 to -1.68	<.001	-1.89	-2.20 to -1.58	<.001
Time from vaccination (month) $t^{-0.5}$	-2.47	-2.95 to -1.98	<.001	-2.36	-2.85 to -1.87	<.001

Vaccination dose	1						
	2	3.08	1.84 to 4.32	<.001	2.67	1.68 to 3.66	<.001
	3	4.86	3.63 to 6.09	<.001	4.23	3.25 to 5.22	<.001
	4	-	-	-	-	-	-

(Supplementary table 3) Regression model for ln (anti-RBD) after COVID-19 vaccination in patients from Group 2

		Univariable			Multivariable	
	coefficient	95% CI	p-value	coefficient	95% CI	p-value
Female sex	0.08	-0.31 to 0.48	0.683	-	-	-
Age (per 10 year)	-0.10	-0.25 to -0.03	0.015	-0.21	-0.30 to -0.11	<.001
BMI	-0.02	-0.10 to 0.05	0.530	-	-	-
DM	0.09	-0.36 to 0.54	0.695	-	-	-
Hypertension	-0.10	-0.45 to 0.26	0.590	-	-	-
History of tuberculosis	-0.36	-1.22 to 0.51	0.419	-	-	-
Chronic liver disease	0.08	-0.40 to 0.55	0.752	-	-	-
Chronic kidney disease	0.75	0.21 to 1.30	0.007	-	-	-
RA duration	-0.01	-0.03 to 0.01	0.359	-	-	-
Glucocorticoid use	-0.19	-0.48 to 0.10	0.205	-	-	-
Prednisolone equivalent	-0.05	-0.11 to 0.00	0.074	-	-	-
Methotrexate	0.09	-0.21 to 0.38	0.577	-	-	-
Hydroxychloroquine	-0.11	-0.41 to 0.20	0.491	-	-	-
Sulfasalazine	0.26	-0.16 to 0.68	0.231	-	-	-
Leflunomide	0.13	-0.26 to 0.52	0.514	-	-	-
Tacrolimus	-0.43	-1.55 to 0.69	0.451	-	-	-
TNF inhibitors	0.42	-0.04 to 0.89	0.075	-	-	-
Tocilizumab	-0.31	-1.33 to 0.72	0.556	-	-	-
Abatacept	-2.85	-3.88 to -1.83	<.001	-3.05	-4.16 to -1.94	<.001
JAK inhibitor	-0.86	-1.46 to -0.27	0.005	-0.93	-1.34 to -0.51	<.001
Rituximab	-2.5	-2.65 to -2.36	<.001	-	-	-
Time from vaccination (month) ln(t)	-0.63	-0.77 to -0.48	<.001	-0.45	-0.61 to -0.28	<.001

	$(\ln(t))^2$	-0.54	-0.65 to -0.44	<.001	-0.47	-0.57 to -0.38	<.001
Vaccination dose	1						
	2	1.19	-0.35 to 2.74	0.129	0.61	-0.33 to 1.56	0.205
	3	2.76	1.25 to 4.27	<.001	1.54	0.62 to 2.47	0.001
	4	3.29	1.68 to 4.90	<.001	1.82	0.69 to 2.94	0.002

(Supplementary Fig. 1) Anti-RBD response to COVID-19 vaccination in patients who used the same type of vaccination

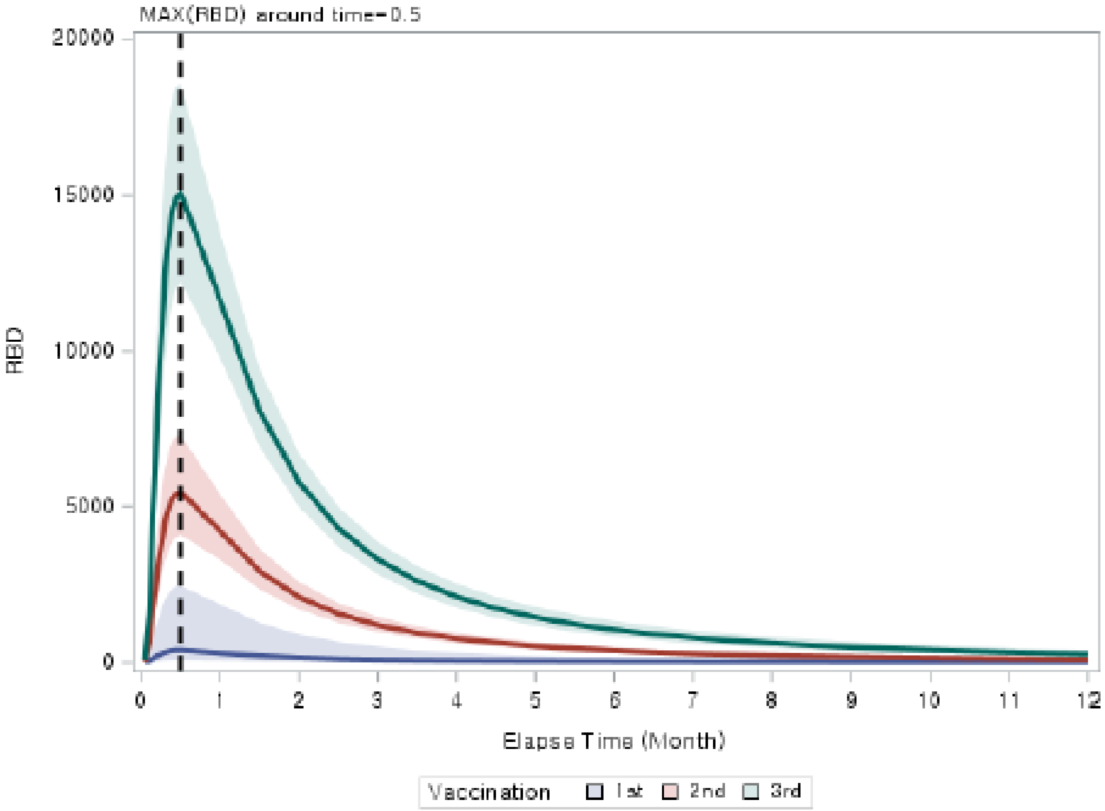

(Supplementary Fig. 2) Anti-RBD response to COVID-19 vaccination in patients from Group 1

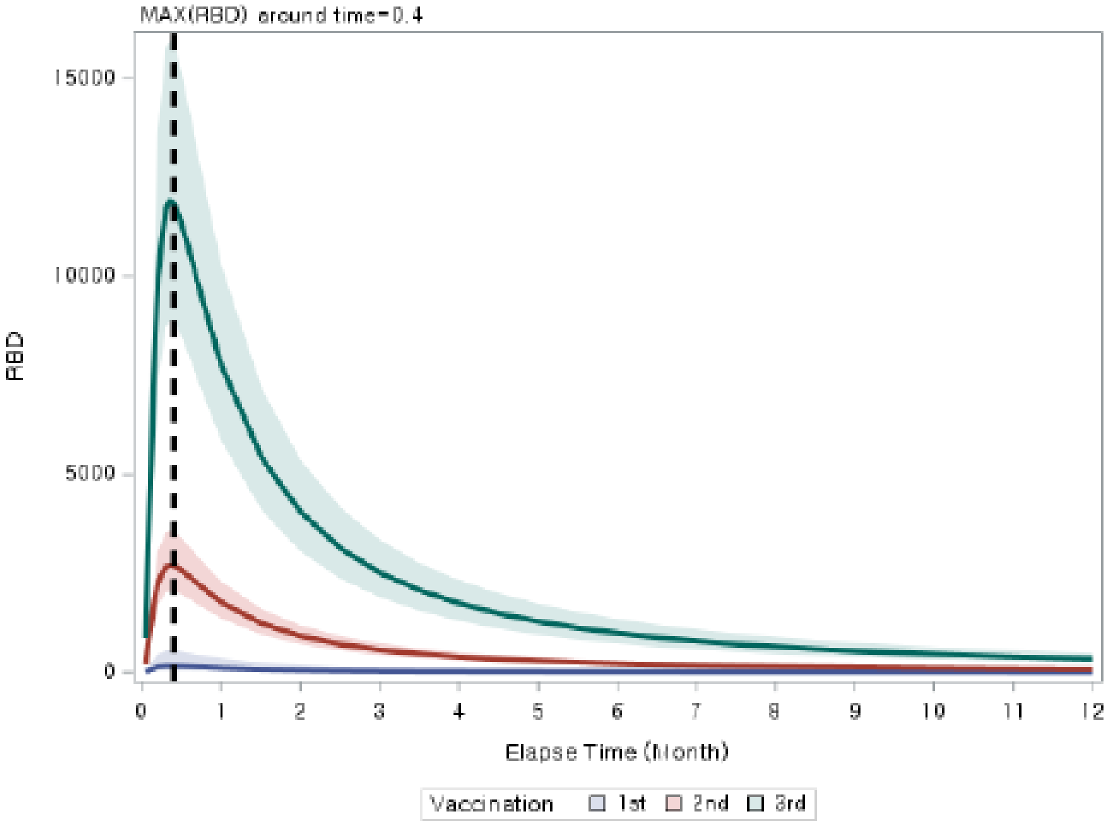

(Supplementary Fig. 3) Anti-RBD response to COVID-19 vaccination in patients from Group 2

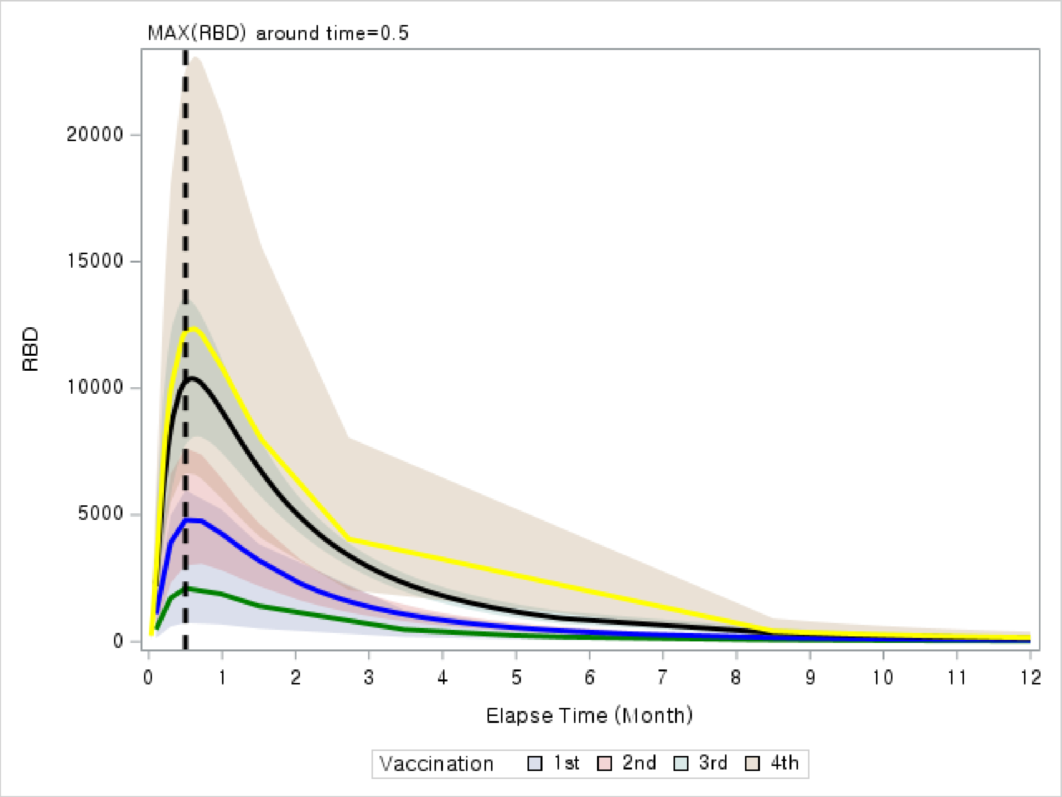

Supplement: Supplementary file 1 — Supplementary Information. [file 41598_2024_51535_MOESM1_ESM.pdf]
